# Sexually dimorphic nuclei in the spinal cord control male sexual functions

**DOI:** 10.3389/fnins.2014.00184

**Published:** 2014-07-11

**Authors:** Hirotaka Sakamoto

**Affiliations:** Laboratory of Neuroendocrinology, Ushimado Marine Institute, Graduate School of Natural Science and Technology, Okayama UniversityOkayama, Japan

**Keywords:** sexual dimorphism, spinal cord, male sexual function, steroid hormones, neuroanatomy

## Abstract

Lower spinal cord injuries frequently cause sexual dysfunction in men, including erectile dysfunction and an ejaculation disorder. This indicates that the important neural centers for male sexual function are located within the lower spinal cord. It is interesting that the lumbar spinal segments contain several neural circuits, showing a clear sexually dimorphism that, in association with neural circuits of the thoracic and sacral spinal cord, are critical in expressing penile reflexes during sexual behavior. To date, many sex differences in the spinal cord have been discovered. Interestingly, most of these are male dominant. Substantial evidence of sexually dimorphic neural circuits in the spinal cord have been reported in many animal models, but major issues remain unknown. For example, it is not known how the different circuits cooperatively function during male sexual behavior. In this review, therefore, the anatomical and functional significance of the sexually dimorphic nuclei in the spinal cord corresponding to the expression of male sexual behavior is discussed.

## Introduction

**Figure 1:**
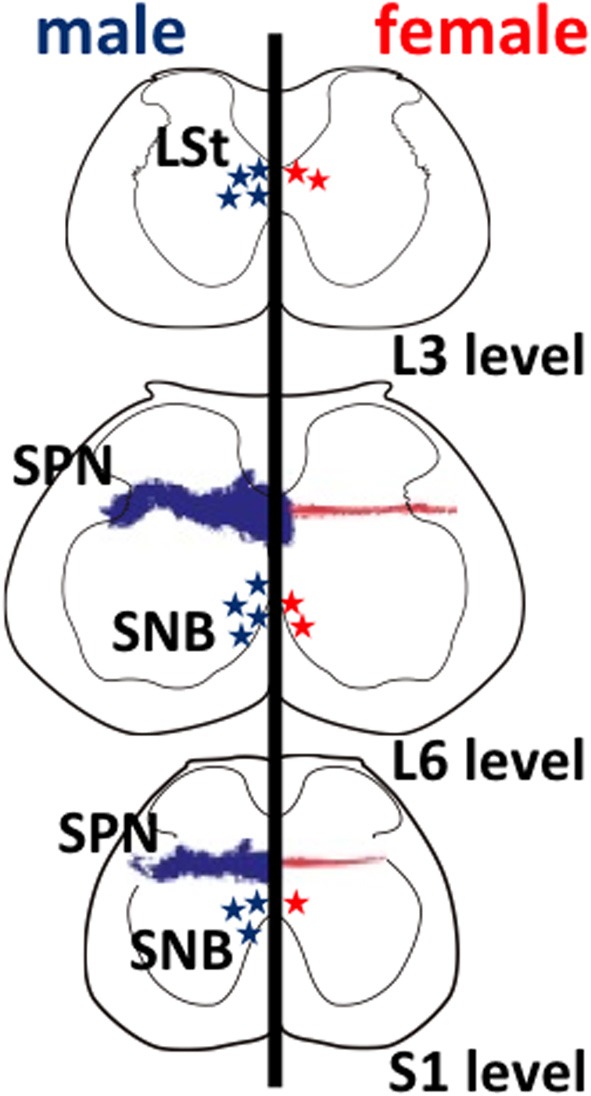
**Schematic drawings of neural sexual dimorphisms in the rodent lumbar spinal cord**. The spinal levels are indicated on the lower right. Anatomical features in males and in females are shown on the left and right hemispheres, respectively. Sexually dimorphic cell numbers and axonal projections in the spinal cord are shown by stars and fine dots (males in blue; females in red), respectively. The density of the symbols is proportional to the relative density of the sexual dimorphisms in the spinal cord. L, lumbar; S, sacral; LSt, lumbar spinothalamic neurons; SNB, spinal nucleus of the bulbocavernosus; SPN, sacral parasympathetic nucleus.

Sexual function and behavior significantly differ in sexes in adulthood, suggesting that the neural circuits also differ between sexes. However, it is difficult to correlate evidence gene expression levels with results of behavioral modifications. It is possible that the sexual differences are affected by a variety of extrinsic and intrinsic factors (Kawata, 1995; Morris et al., 2004; Sakamoto, 2012). Masculine sexual behavior is complex and modulated by intrinsic as well as extrinsic factors, including sensory inputs, autonomic regulations and their circumstances (Rosen and Sachs, 2000; Coolen, 2005). Spinal cord injuries located at the lower levels frequently cause sexual dysfunction in men, including erectile dysfunction and an ejaculation disorder (Sipski, 1998; Brown et al., 2006). This indicates that the important neural centers for male sexual function are located within the lower spinal cord. It is likely that the lumbar spinal segments contain several neural circuits, showing a clear sexually dimorphism that, in association with neural circuits of the thoracic (sympathetic) and sacral (parasympathetic) spinal cord. They play an important role in eliciting penile responses (i.e., erection and ejaculation) (Breedlove and Arnold, 1983a,b; Breedlove, 1985; Morris et al., 2004; Matsuda et al., 2008; Sakamoto, 2012) (see Figure 1). Substantial evidence of sexually dimorphic neural circuits in the spinal cord have been reported in many animal models, but major issues remain unknown, such as how they cooperatively function during male sexual behavior. In this review, the anatomical and functional significance of the sexually dimorphic nuclei in the spinal cord corresponding to the expression of male sexual behavior is discussed.

## Lumbar spinothalamic (LSt) neurons

Several studies have identified that a male-dominant sexual dimorphism in the lumbar spinal cord is observed in rats (Figure 1). These neurons are located within the third and fourth lumbar segments of the spinal cord dorsolateral to the central canal in lamina X and express galanin (Newton, 1992), cholecystokinin (Phan and Newton, 1999), and enkephalin (Nicholas et al., 1999), possibly projecting to medial portion of the parvocellular subparafascicular thalamic nucleus (mSPFp) (Ju et al., 1987; Truitt et al., 2003). Therefore, they are a male-dominant sexually dimorphic nucleus, and so-called lumbar spinothalamic (LSt) neurons (Ju et al., 1987; Truitt et al., 2003). In rats, the increased Fos expression can be considered as a marker for neural activation. The activation of these LSt neurons is triggered by stimuli associated with ejaculation specifically, but mounts or intromission did not trigger Fos expression in LSt neurons. It is suggested that a specific subpopulation of LSt neurons signals information associated with ejaculation in rats (Truitt et al., 2003). A specific population of LSt neurons in the lumbar segments (L3–L4 level) of the spinal cord acts as a “spinal ejaculation generator” because ablation of these neurons by the selective toxins resulted in a complete disruption of ejaculatory behavior in rats (Truitt and Coolen, 2002). In contrast, other components of male sexual behavior remain intact, suggesting that this population of LSt neurons plays an important role in generation of ejaculation and is part of a spinal ejaculation generator (Truitt and Coolen, 2002). Furthermore, these LSt neurons convey the sexual information to the thalamus (Ju et al., 1987; Truitt et al., 2003), and integrate the information within the neural connections between LSt and autonomic/somatic centers in the spinal cord (Xu et al., 2005, 2006; Sun et al., 2009). However, the central and molecular mechanisms, including the neuropeptides involved, that directly regulate erection and ejaculation remain unclear.

## The gastrin-releasing peptide (GRP) system in the lumbar spinal cord

**Figure 2:**
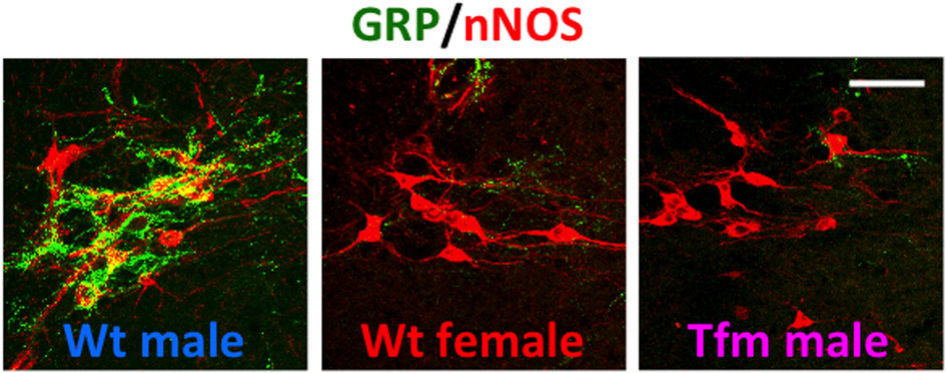
**A newly discovered sexual dimorphism in the lumbar spinal cord of rats that controls male sexual function**. Co-immunofluorescence for gastrin-releasing peptide (GRP) (green) and neuronal nitric oxide synthase (nNOS) (red) confirmed that GRP-containing axons surrounding the autonomic sacral parasympathetic nucleus (SPN) neurons are significantly prominent in male rats, but vestigial or absent in females. In addition, testicular feminization mutation (Tfm) male rats display an entirely feminine pattern of GRP-containing fibers in the SPN autonomic nucleus. Wt, wild-type. Scale bar, 50 μm. The figure was reproduced from Sakamoto et al. (2008) with permission.

Using immunohistochemistry for gastrin-releasing peptide (GRP) in rats, we newly identified a collection of neurons containing GRP in the lumbar spinal region (L3–L4 level), showing a clear male-dominant sexual dimorphism (Sakamoto et al., 2008; Sakamoto, 2011) (Figure 2). These GRP-expressing neurons send axons onto the more caudal segments of the lumbosacral spinal cord (L5–L6 and S1 levels), including the autonomic sacral parasympathetic nucleus (SPN) as well as the somatic spinal nucleus of the bulbocavernosus (SNB) (Sakamoto et al., 2008). Double immunofluorescence of GRP and neuronal nitric oxide synthase (nNOS), a marker for autonomic preganglionic (SPN) neurons (Vizzard et al., 1995) clearly showed that GRP-expressing fibers densely projected into the SPN (Sakamoto et al., 2008). These GRP-expressing fibers surrounding cell bodies and dendrites of the SPN neurons were observed in only males but vestigial or absent in females (Sakamoto et al., 2008) (Figure 2). It is reported that the autonomic SPN neurons play a pivotal role in controls of penile function and express high levels of nNOS (Studeny and Vizzard, 2005). In rats, these GRP neurons located in the lumbar spinal cord also express androgen receptor (AR) but do not express estrogen receptor alpha subtype (ERα) (Sakamoto et al., 2008; Sakamoto and Kawata, 2009). Using genetically male (XY) rats carrying the testicular feminization mutation (*Tfm*) of the *AR* gene, we examine whether androgens direct sexual differentiation of these GRP neurons. These mutant males develop testes embryologically and secrete testosterone prenatally. However, their AR protein is dysfunctional, they develop a complete feminine exterior phenotype, including a clitoris rather than a penis. We found that the spinal cord of genetic male rats, carrying the *Tfm* allele for *AR* hyperfeminine characteristics, have even fewer GRP-positive neurons in this region than do wild-type females (Figure 2). In this species, GRP-containing presynaptic boutons have also been shown by electron microscopic immunohistochemistry to innervate nNOS-positive dendrites in the autonomic SPN of the lower lumbar and upper sacral spinal cord (Sakamoto et al., 2008).

Substantial evidence indicated that the presence of a GRP receptor (GRPR) in the lumbar and sacral spinal cord of rats based on specific binding of GRP (Sakamoto et al., 2008). The higher expressions of GRPR at the mRNA and protein levels in SPN neurons were also obvious from the immunochemical and PCR analyses in rats (Sakamoto et al., 2008, 2009). Furthermore, the rat homolog of GRPR agonists (rGRP_20−29_) (Ladenheim et al., 1996) is able to restore a lot of the spinal reflexes of the penis that are lost after orchiectomy (Sakamoto et al., 2008). The agonists were mostly effective in reinstating ejaculatory reflex *per se*, resulting in a greater frequency of ejaculation in treated castrates than in gonadally intact control males (Sakamoto et al., 2008). To probe whether GRPR activation of penile reflexes is mediated by the spinal cord, we also administered RC-3095, a specific GRPR antagonist (Pinski et al., 1992; Roesler et al., 2004), intrathecally to the lumbosacral spinal cord of gonadally intact males. The antagonistic treatment significantly inhibited penile reflexes, including simple erections, dorsal flips of the penis, and cup-like flaring erections of the distal glans, and also attenuated the spontaneous ejaculation rate (Sakamoto et al., 2008). These results indicate that the GRP/GRPR system controlling masculine reproductive function is within the lower spinal cord (Sakamoto et al., 2008).

The identification of this male-specific neural system using a specific neuropeptide, GRP, that controls sexual function offers new approaches for the development of pharmacological treatments to relief the male reproductive dysfunction (Sakamoto, 2011). In addition to the parasympathetic nucleus, LSt neurons in the spinal cord project to the sympathetic neurons of the intermediolateral column in the thoracic spinal cord, which is crucial for the emission phase of ejaculation (Coolen, 2005; Kozyrev et al., 2012). The local administration of the specific blocker for GRPR, RC-3095 significantly attenuated bursts in response to dorsal penile nerve stimuli of the bulbocavernosus muscles that are innervated by the SNB motoneurons during ejaculation (Kozyrev et al., 2012). This supports our hypothesis that GRP in the spinal cord plays a pivotal role in the regulation of penile reflexes during masculine copulatory behavior in rodents. Clinically, the next question is now: Does the spinal GRP system exist and function in the human spinal cord? Future attention should be focused on comparative studies for the spinal GRP system using other vertebrates, including humans and/or primates.

## Onuf's nucleus (SNB)

**Figure 3:**
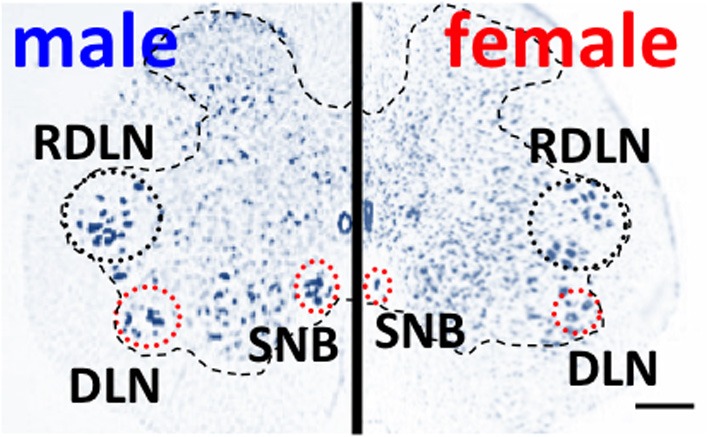
**Spinal nucleus of the bulbocavernosus (SNB) motoneurons are more numerous in male than in female rats**. The dorsolateral nucleus (DNL) is also male-dominant sexually dimorphic, but the retrodorsolateral (RDLN) nucleus is similar in both male and females. Scale bar, 200 μm.

Onufrowicz (1899) reported that a sexually dimorphic nucleus is located in the motor pools of the sacral spinal cord in most mammals, that innervates the penile functions involved in sexual behavior; so-called Onuf's nucleus. In humans, Onuf's nucleus are composed of a discrete group of motoneurons located in the ventral motor pool of the sacral spinal cord that play an important role in the micturition and defecatory as well as in rhythmic contractions of perineal muscles during orgasm (Onufrowicz, 1899). The number of motoneurons in Onuf's nucleus in humans is a sexually dimorphic: greater in men than that in women (Onufrowicz, 1899; Sato et al., 1978; Nakagawa, 1980; Forger and Breedlove, 1986). In rats, the SNB is located in the lumbosacral spinal cord; it is homologous to Onuf's nucleus in humans in that it innervates the striated perineal muscles attached to the base of the penis (Breedlove and Arnold, 1980; Forger and Breedlove, 1986; Sengelaub and Forger, 2008) (Figures 1, 3). Although the dorsolateral nucleus (DLN) innervating the ischiocavernosus and external urethral sphincter is also sexually dimorphic, the retrodorsolateral (RDLN) motoneurons that innervate foot muscles show no sexual difference and are relatively unresponsive to androgens (Jordan et al., 2002; Ottem et al., 2007) (Figure 3). Male rats have more and larger SNB as well as DLN motoneurons than females, a dimorphism that results from differences in perinatal androgen signaling through an AR-mediated mechanism (Breedlove and Arnold, 1980) (Figure 3). This male-dominant sex difference in SNB first found in rats has been extended to many mammalian species, including mice, cats, gerbils, dogs, hyenas, and monkeys (Ueyama et al., 1984, 1985; Forger and Breedlove, 1986; Wee et al., 1988; Ulibarri et al., 1995; Forger et al., 1996). The sex-related difference in the number of SNB motoneurons develops perinatally in rats. Prior to birth, the number of motoneurons in the SNB increases and reaches a maximum in both sexes; at this time, functional neuromuscular junctions have been established in the SNB system. However, in female rats, these components (both motoneurons and muscles) die near the time of birth unless the animals are exposed to testosterone during the critical period (androgen surge) (Nordeen et al., 1985). If an androgen surge occurs, it results in higher expression of AR in both the perineal muscles and spinal motoneurons. In male rats, testosterone primarily prevents the muscle from dying, which secondarily prevents the death of motoneurons in the spinal cord. Testosterone is thought to induce the muscle to produce a neurotrophic factor that protects the muscle, and the same factor or an additional factor then protects the motoneurons from developmental cell death. However, it is unclear which downstream genes first respond to testosterone. In the SNB system, testosterone may alter the expression of trophic factor genes to spare both the muscle and innervating motoneurons. Ciliary neurotrophic factor (CNTF) is a candidate trophic factor for the perineal neuromuscular junction because receptors for CNTF are expressed in the motoneurons and in their target muscles (Davis et al., 1991; Ip et al., 1993), and injection of CNTF into the perineum of newborn rats spares the SNB system in normal females (Forger et al., 1993).

## Interaction of the GRP system with both the autonomic and somatic nuclei in the spinal cord

Orchiectomy of adult male rats results in the shrinkage of soma size and dendritic arborization of SNB motoneurons as well as in a reduction in the number of synaptic inputs, all of which can be prevented by testosterone replacement for castrates (Breedlove and Arnold, 1981; Kurz et al., 1986; Matsumoto et al., 1988; Goldstein et al., 1990; Yang et al., 2004). In female rats, a long-term testosterone treatment to castrates also increase the SNB motoneuronal cell size, however, the increase did not reach to the level observed in males (Breedlove and Arnold, 1981; Sengelaub and Forger, 2008). The GRP system in the lumbosacral spinal cord also showed similar results; regarding the lower sensitivity to androgens in adult females (Sakamoto et al., 2008, 2009) and the AR signaling cascades that are necessary to maintain the GRP and SNB systems in the lumbosacral spinal cord in adult males (Sengelaub and Forger, 2008; Forger, 2009; Sakamoto and Kawata, 2009). It is interesting that the GRP and SNB systems, which are localized at the same lower spinal cord, might interact directly or indirectly to modulate the male sexual function. Furthermore, the sexually dimorphic distribution of GRP-containing fibers in the lumbosacral spinal cord (L5–L6 and S1) is controlled by circulating testosterone levels (Sakamoto et al., 2009) and mirroring changes in SNB motoneurons in male rats (Kurz et al., 1986; Matsumoto et al., 1988; Matsumoto, 2001). Recently, it has been reported using a mouse line specifically lacking *AR* in the nervous system (AR^NesCre^) that the number of SNB motoneurons is unrelated to both AR^NesCre^ mutation status (Raskin et al., 2012), although adult AR^NesCre^ males exhibit higher levels of circulating testosterone than controls (Raskin et al., 2009). In the SNB, the central AR participates in the developmental regulation of both soma size and dendritic length but not in the survival of SNB motoneurons (Raskin et al., 2012). Immunohistochemical studies in the lumbosacral spinal cord also demonstrated the expression of AR in the cellular nuclei of SNB motoneurons in controls but not in AR^NesCre^ males (Raskin et al., 2012). However, loss of *AR* expression in the nervous system caused a significant decrease in the number of GRP-immunoreactive neurons compared with that in control littermates (Sakamoto et al., 2014). Consequently, the intensity of GRP axonal projections to the lower spinal cord (L5–L6 and S1 level) was greater in control males than that in AR^NesCre^ males (Sakamoto et al., 2014). Taken together, these results suggested that nervous system AR participates in both morphological differentiation and adult activation of SNB motoneurons, but not directly in the survival of SNB motoneurons during neonatal development (Raskin et al., 2012). In contrast, ARs expressed in the nervous system play critical roles in the development as well as in the maintenance of GRP neurons in the lumbosacral spinal cord in males. The *AR*-deletion mutation may attenuate sexual behavior and activity of mutant males *via* spinal GRP system-mediated neural mechanisms (Raskin et al., 2009; Sakamoto et al., 2014).

High-voltage electron microscopy (HVEM) is a powerful methodology for studying chemical neuroanatomy at the ultrastructural level, and the results with this method can be easily linked to the conventional light and electron microscopies (Sakamoto and Kawata, 2012). We combined an immunohistochemistry with a retrograde labeling technique utilizing a cholera toxin beta subunit-horseradish peroxidase conjugate under the HVEM. Three-dimensional (3-D) analysis by HVEM provided clear solid visualization of synaptic contacts from the spinal GRP system to the SNB motoneurons in male rats (Sakamoto et al., 2010; Sakamoto and Kawata, 2012). By means of a double labeling with immunohistochemistry and retrograde tracing, we observed that the many GRP-immunoreactive axons directly contact dendrites of the SNB motoneurons on a single section (Sakamoto et al., 2010; Sakamoto and Kawata, 2012). The molecular and neural regulations of male sexual behavior by the GRP system at the spinal cord level are revealed by HVEM at the 3-D ultrastructural level (Sakamoto et al., 2010; Sakamoto and Kawata, 2012). Because the bulbocavernosus muscles are considered to be a homologous to Onuf's nucleus in humans, they play an important role in the rhythmic contractions of perineal muscles during ejaculation also in rats (Sachs, 1982). Therefore, these 3-D results taken together suggested that GRP-containing afferents to SNB motoneurons may control penile reflexes during sexual behavior through the identified GRP-SNB synapses (Sakamoto et al., 2010). Nevertheless, the functional synchronization of these two neural systems in the lower spinal cord is required for normal penile reflexes (Sakamoto, 2011). Using HVEM, we further demonstrated that the terminals of GRP neurons may form 3-D multiple synapses with the dendrites of SPN neurons revealed by a double immunohistochemical study (Oti et al., 2012). Using a viral trans-synaptic retrograde tracing technique, Dobberfuhl et al. (2014) recently reported that after the pseudorabies virus (PRV) injection into the levator ani muscle, about a half of PRV-positive neurons in the medial gray at the upper lumbar spinal cord level expressed GRP. Interestingly, very few PRV-labeled spinal interneurons were found in the medial region of the upper lumbar spinal cord in preadolescent pups. These results indicate the presence of either direct or indirect synaptic contacts from GRP-containing neurons to SPN (autonomic) neurons and/or to SNB (somatic) motoneurons, and these neural circuits might develop during puberty. It has also been reported that GRPRs are expressed in both the SPN and SNB (Sakamoto et al., 2008). Thus, a spinal GRP/GRPR system could generate an ejaculatory behavior by synchronizing autonomic and somatic centers; e.g., the SPN and SNB in the lumbosacral spinal cord. A set of these findings supports the hypothesis that the GRP/GRPR system may regulate male sexual behavior *via* afferents to both SPN and SNB neurons, and coordinate autonomic and somatic functions in response to penile reflexes during male copulatory behavior.

## Afferents from the spinal GRP system to the brain

Truitt and Coolen (2002) reported a potential ejaculation generator in the spinal cord in rats. Because LSt neurons project to the brain thalamus and are involved in the relay of ejaculation-related sensory information and/or sexual arousal to evoke ejaculation, the discovery of the “spinal ejaculation generator” provides an excellent target for further understanding of the neural processes controlling ejaculatory behavior. Namely, the characterization of hormonal dynamics involved in the modulation of either LSt neuronal function or the activation of LSt neuronal target cites is required for a better understanding of the molecular mechanisms underlying the expression of male sexual behavior. GRP and galanin might be possible candidates for neuromodulator(s) regulating LSt neuronal activity (Truitt et al., 2003; Sakamoto et al., 2008). In fact, local injection of galanin into the mSPFp significantly attenuated male copulatory behavior in rats (Coolen, 2005), suggesting that LSt signaling might play an important role in the refractory period after ejaculation. On the other hand, similar microinjection of galanin into neighboring thalamic areas did not affect any components of male sexual behavior (Coolen, 2005). Since the detailed molecular mechanisms of ejaculatory behavior in the central nervous system remains unknown, further investigation of the LSt-mSPFp interaction is required to draw a firm conclusion.

## Conclusions

Further understanding of the neural and molecular basis of the sexual dimorphism in the central nervous system will progress our understanding of the expression of the sexually different behavior. The expression of sexual behavior in vertebrates is properly affected by the interactions between endocrine and psychological factors. During the ontogeny, therefore, it is important to know how, when, and where sex steroid hormones (estrogens and/or androgens) behave in the sexual differentiation of the brain and spinal cord *via* the genomic and/or non-genomic actions. The sexually differentiated nervous system is influenced by the region- and temporal-specific sex steroid milieu, suggesting a significance of sex differences observed in many neurobiological dysfunctions. Although this agenda is especially difficult and controversial when applied to humans because of the highly social species, interdisciplinary studies at the molecular, behavioral, and social levels might be able to make demonstration of the hormonally orchestrated sexual dimorphism in the nervous system and related clinical disorders in humans.

### Conflict of interest statement

The author declares that the research was conducted in the absence of any commercial or financial relationships that could be construed as a potential conflict of interest.
